# Compound-specific isotope analysis of diesel fuels in a forensic investigation

**DOI:** 10.3389/fchem.2015.00012

**Published:** 2015-02-27

**Authors:** Syahidah A. Muhammad, Russell D. Frew, Alan R. Hayman

**Affiliations:** ^1^Department of Chemistry, University of OtagoDunedin, New Zealand; ^2^Environmental Technology Division, School of Industrial Technology, Universiti Sains MalaysiaPulau Pinang, Malaysia; ^3^Doping Control Centre, Universiti Sains MalaysiaPulau Pinang, Malaysia; ^4^Division of Nuclear Applications in Food and Agriculture, International Atomic Energy AgencyVienna, Austria

**Keywords:** diesel fuel, alkane, compound-specific isotope analysis (CSIA), principal component analysis (PCA), hierarchical clustering analysis (HCA)

## Abstract

Compound-specific isotope analysis (CSIA) offers great potential as a tool to provide chemical evidence in a forensic investigation. Many attempts to trace environmental oil spills were successful where isotopic values were particularly distinct. However, difficulties arise when a large data set is analyzed and the isotopic differences between samples are subtle. In the present study, discrimination of diesel oils involved in a diesel theft case was carried out to infer the relatedness of the samples to potential source samples. This discriminatory analysis used a suite of hydrocarbon diagnostic indices, alkanes, to generate carbon and hydrogen isotopic data of the compositions of the compounds which were then processed using multivariate statistical analyses to infer the relatedness of the data set. The results from this analysis were put into context by comparing the data with the δ^13^C and δ^2^H of alkanes in commercial diesel samples obtained from various locations in the South Island of New Zealand. Based on the isotopic character of the alkanes, it is suggested that diesel fuels involved in the diesel theft case were distinguishable. This manuscript shows that CSIA when used in tandem with multivariate statistical analysis provide a defensible means to differentiate and source-apportion qualitatively similar oils at the molecular level. This approach was able to overcome confounding challenges posed by the near single-point source of origin, i.e., the very subtle differences in isotopic values between the samples.

## Introduction

Compound-specific isotopic analysis (CSIA) is a technique that is becoming increasingly popular as a forensic tool to measure stable isotope composition of chemical compounds, such as hydrocarbons, which can be likened to fingerprinting at the molecular level. From a forensic perspective, it is important to infer a link between a sample to a suspected source(s) by obtaining chemical information from both data sets or to differentiate the sample in question from the other samples of known origin, i.e., a population study where an informed assessment can be made by obtaining the profiles of the sample population to provide context for the data. In other words, stable isotope fingerprinting provides the resolution to the question of whether two compounds or substances are distinguishable, i.e., un-related. If the two samples are indistinguishable this supports the hypothesis that they are related. Although variations in stable isotope ratios are generally very small they are robust and have allowed workers in numerous fields to formulate links between samples (e.g., Brand and Coplen, 2012). With the help of stable isotope fingerprinting, forensic scientists are able to support inferences to link a person to an event, a crime scene, or a criminal organization, based on a unique characteristic of some physical evidence. Traceability of diesel fuel, i.e., to demonstrate any linkages, or relationships, between diesel samples is very important to ascertain culpability in a diesel theft case. Therefore, to have methods to unambiguously characterize, identify and assign sources is key to withstand the legal scrutiny in the court of law.

Current techniques for the characterization of oil products generally use gas chromatography (GC) to separate and quantify the different molecular species. Crude oils from different sources exhibit different characteristics in terms of the ratios of molecular species. Processing of the oil changes these characteristics, e.g., the high molecular weight pentacyclic terpenes and steranes are generally removed during the refining process whereas the diamondoids (adamantanes and diamantanes) are found in most petroleum products (Wang et al., 2006). Recent advances in the interfacing of GC to isotope ratio mass spectrometers offers the potential to enhance the fingerprinting capability of the GC techniques by harnessing the discriminating power offered through isotope ratio measurements (Turner et al., 2006). Petroleum-derived hydrocarbon samples of different origin and/or history have been shown to be distinguishable by CSIA based on the stable isotope signatures albeit the differences obtained are subtle (Philp et al., 2002; Smallwood et al., 2002).

Petroleum products are generally mixtures of volatile, semi-volatile and refractory compounds. These will exhibit quite different isotopic evolution during processing and/ degradation with some compounds changing very little and others exhibiting very significant change (e.g., Muhammad et al., 2015). Furthermore, once leaving the refinery or following release into the environment, the original isotopic signatures may change due to fractionation or mixing processes. This tremendous isotopic variability offers a strong forensic tool in comparing/contrasting different samples of the same compound (contaminant). Thus, careful selection of target molecules offers the means to identify the origin of the petroleum products.

A few studies have incorporated multivariate statistics to correlate and differentiate petroleum hydrocarbons to its source(s) using stable isotope fingerprints (Boyd and Coffin, 2004; Boyd et al., 2006). These statistical techniques are suitable to be applied in the area of environmental hydrocarbon fingerprinting due to the large number of samples and variables involved. Principal component analysis (PCA) is an exploratory statistical analysis which is frequently applied in this area due to its ability in detecting potential group tendency within a sample set; i.e., to assign a class membership to each sample. PCA can also reveal underlying features in the dataset that are responsible for the detected classification (Pasadakis et al., 2008). Another statistical technique which is also frequently utilized in oil-source correlation work is hierarchical clustering analysis (HCA). Models are built based on distance connectivity between samples in a multidimensional space spanned by the original variables in this type of analysis. The objective is to assign each sample to a group of objects in a stepwise manner where extensive hierarchies of clusters merge with each other at certain distances (Muhammad et al., 2013).

The objective of this study is to carry out forensic fingerprinting of diesel fuels based on the isotopic character of a suite of hydrocarbon compounds as diagnostic indices. The relationship between samples in the data set was then explored using multivariate statistical analyses to provide a defendable means of classification.

## Materials and methods

### Sample details and preparation

**Figure 1:**
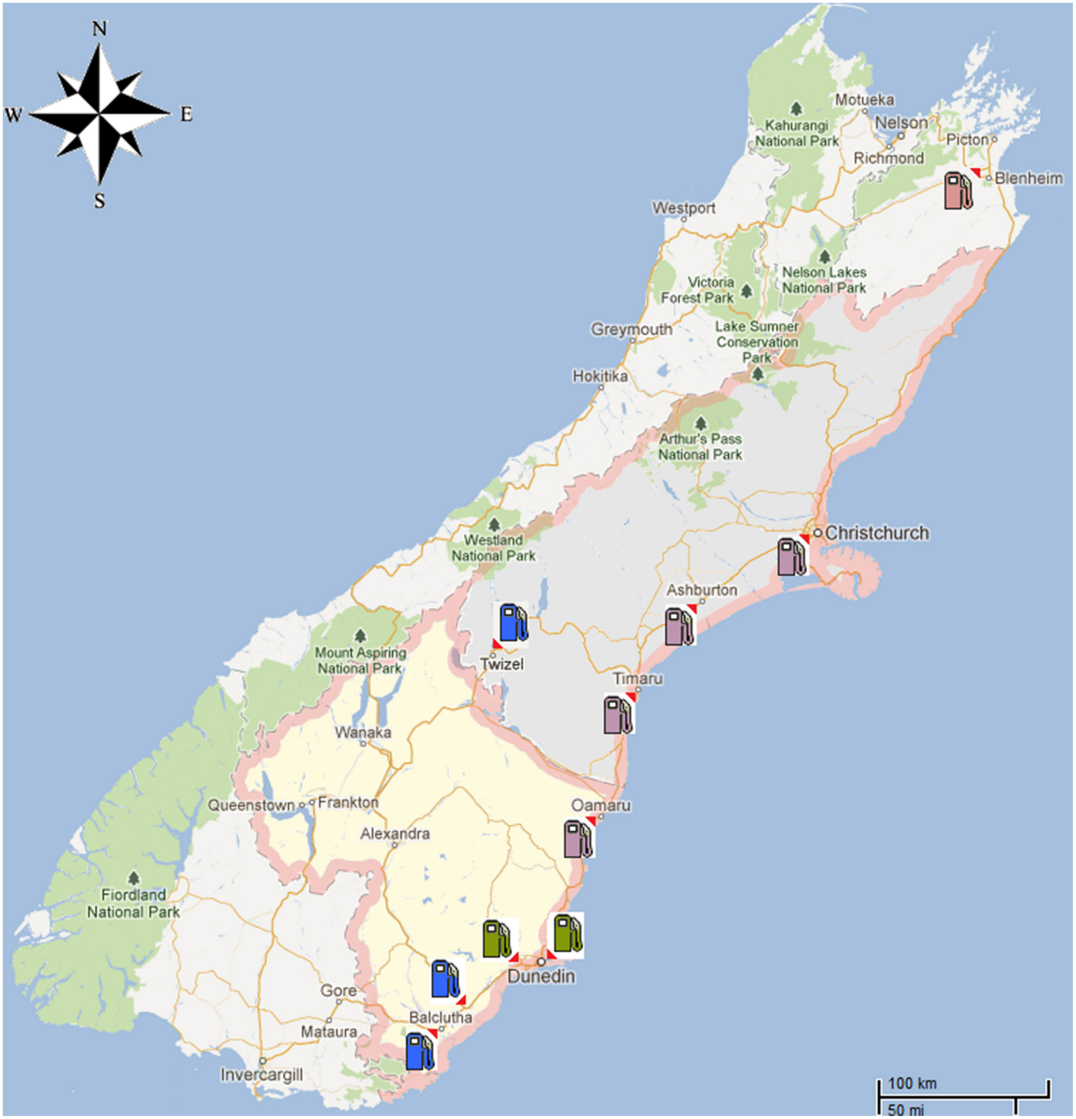
**A map of the South Island of New Zealand showing the diesel theft location as well as the area where the 45 diesel samples were obtained from commercial service stations**. Locations were marked as follows: Blenheim (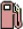), Dunedin City (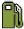), Canterbury region and North Otago (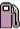), South Otago and Twizel (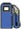).

Case samples were provided by the Blenheim Police Department and arrived in our laboratory in separate, labeled bottles with polypropylene screwed caps. The samples were labeled in two different categories: (1) Control (2 samples); (2) GKT ### (9 samples). The Control samples were taken from the storage tanks located at the ski field in Blenheim (Figure 1) where the diesel theft occurred and the GKT samples with different denominations were sub-sampled from containers found with the suspect. The diesel from the ski field was winterized diesel. This type of diesel is formulated to withstand the freezing temperature during the winter to avoid it coagulating and becoming solid in fuel lines. Fluidity was enhanced by adding a small amount of additive (i.e., lower molecular weight hydrocarbons) to improve liquefaction. In this regard, these winterized diesels would possibly show distinct fingerprints from the regular diesels found at service stations. No information was provided as to the origin of the samples or, e.g., whether the samples were replicates as the sampling details were kept secret according to the provisions of law.

To provide context for the analysis of the diesel fuel involved in the theft case, a further 45 commercial diesel samples were obtained from different service stations located around the South Island of New Zealand (Figure 1). These samples came from areas such as Dunedin City, North and South Otago, Twizel, Christchurch City and South Canterbury (Muhammad et al., 2013).

The diesel fuels were prepared for GC analysis by sub-sampling 20 μL of each sample and dispensed using a micro syringe into a GC vial which was then diluted to 2 mL with *n*-pentane. All samples were prepared in duplicate.

### GC analysis

Gas chromatographic and isotope analyses were obtained on at least two aliquots of each sample and each analysis was an average of at least three measurements. Compound specific carbon and hydrogen isotope ratios were determined using a Trace Ultra-gas chromatograph (Thermo, Milan, Italy) coupled to a Delta^plus^ XP isotope ratio mass spectrometer (Thermo, Bremen, Germany) via a high temperature conversion furnace, heated to 940°C and 1450°C for carbon and hydrogen analyses, respectively. The injection mode used was splitless with the temperature set at 300°C. Compounds were separated using a HP-1 GC column (30 m, 0.32 mm i.d., 0.25 μm film thickness: J&W scientific). Identification of the analytes was made prior to isotope analysis using GC-FID by comparing the retention time of DRH-008S-R2 hydrocarbon standard solution (AccuStandard, Connecticut, USA), which contains 35 *n*-alkane compounds in chloroform, with that of the samples. The GC analytical conditions for both instruments were set to be the same throughout each run to avoid misrepresentation. The carrier gas for the analysis was helium with a constant flow of 1.5 ml/min. The parameters for the analytical run were as follows: initial oven temperature 50°C, initial hold time of 1 min, temperature ramp at 10°C min to 300°C, and final hold time 4 min.

Instrumental drift during individual sample analysis (~30 min) was corrected by injecting multiple pulses of monitoring gas (CO_2_ or) at the beginning and end of each sample run. While instrumental drift during a batch sample was corrected by injecting a standard mixture (containing seven *n*-alkanes whose δ ^13^C and δ^2^H had been previously determined by bulk IRMS) every six samples. The instrument analytical precision for compound specific δ ^13^C and δ^2^H analysis was determined to be <0.3 and 3%, respectively, based on long-term repeatability of a control sample. Isotopic compositions of each alkane were expressed as δ values per mil (%) deviation relative to isotopic standard reference materials:

(1) $$\delta =[(R_{sample}/R_{standard})-1]$$

where *R* = ^13^C/^12^C or ^2^H/^1^H. The δ^13^C were reported relative to the Vienna Pee Dee Belemnite (VPDB), while δ^2^H values were reported relative to the Vienna Standard Mean Ocean Water (VSMOW) standard. The H^+^_3_ factor was determined daily using the standard hydrogen gas introduced through the interface. The H^+^_3_ is a byproduct species formed during ion-molecule reaction which can interfere with the measurement of HD, so correction was required for H isotope determination. The mass spectrometer was tuned to ensure that the H^+^_3_ factor was less than 10 nA/ppm and the daily variability was <0.1.

### Isotopic composition of alkanes in diesel fuel

Diesel fuel is a complex mixture of hydrocarbon molecules derived from petroleum crude oil and may contain thousands of individual compounds, most with carbon numbers between 9 and 23. This complexity can cause analytical problems in gas chromatography such as peak separation and baseline resolution, even more so with GC-IRMS as true compound-specific isotopic analysis requires baseline resolution and no co-eluting peaks. Hence, it is important to note that the measured stable isotope ratios across selected peaks may therefore include some underlying co-eluting material and are not entirely specific for individual compounds. However, the data in this study remain forensically relevant as part of an “isotopic fingerprint pattern.” The alkane compounds which were able to be reliably quantified and yielded reproducible isotopic values using a GC-IRMS system in this study started with *n*C_12_ and ended with *n*C_23_.

### Data treatment using statistical methods

All mathematical and statistical computations were made using Excel 2007 (Microsoft Office®), SigmaPlot 11.0 (Systat Software Inc.®) and SPSS 16.0 (IBM®). Multivariate statistical analysis of the stable isotope data was performed using PCA and HCA.

PCA is a mathematical procedure that converts the possibly correlated original variables into new linearly uncorrelated variables, called the principal components (PCs). This technique also reveals the internal structure of the data and finds the indices which best explains the variance in the data set. PCA also provides the most meaningful parameters which interpret the whole data set, thus reducing the dimensionality of the transformed data and summarize the statistical correlation among constituents with minimum loss of original information (Kazi et al., 2009). For the purpose of this study, PCA was used as an exploratory technique to group samples with similar isotopic compositions in PC space. The outcomes of the analysis can be visualized using scores plot which illustrate the tendency of a sample grouping. The contribution of certain characteristics which is called patterns or loadings of the data set will determine the position of each sample in the scores space (Pasadakis et al., 2008).

HCA is an analysis that builds a hierarchy of clusters by measuring either the distance or the similarity between the objects to be clustered. It is normally used when there are no *a priori* hypotheses. The hierarchical agglomerative clustering or the “bottom up” method is the preferred approach for applying this technique. Briefly, it builds the hierarchy from the individual elements by progressively merging clusters. The results of HCA are normally illustrated using a dendrogram (tree diagram). The dendrogram summarizes the clustering process, showing the number of clusters (number criterion) and indicating their proximity in space (distance criterion), thus reducing the dimensionality of the original data. To analyze the data set in this study, HCA was performed using Ward's linkage method. The Ward's method minimizes the total within-cluster variance, and the cluster distances are defined by the squared Euclidean distances as a measure of similarity.

## Results and discussion

### Carbon isotope analysis

**Figure 2:**
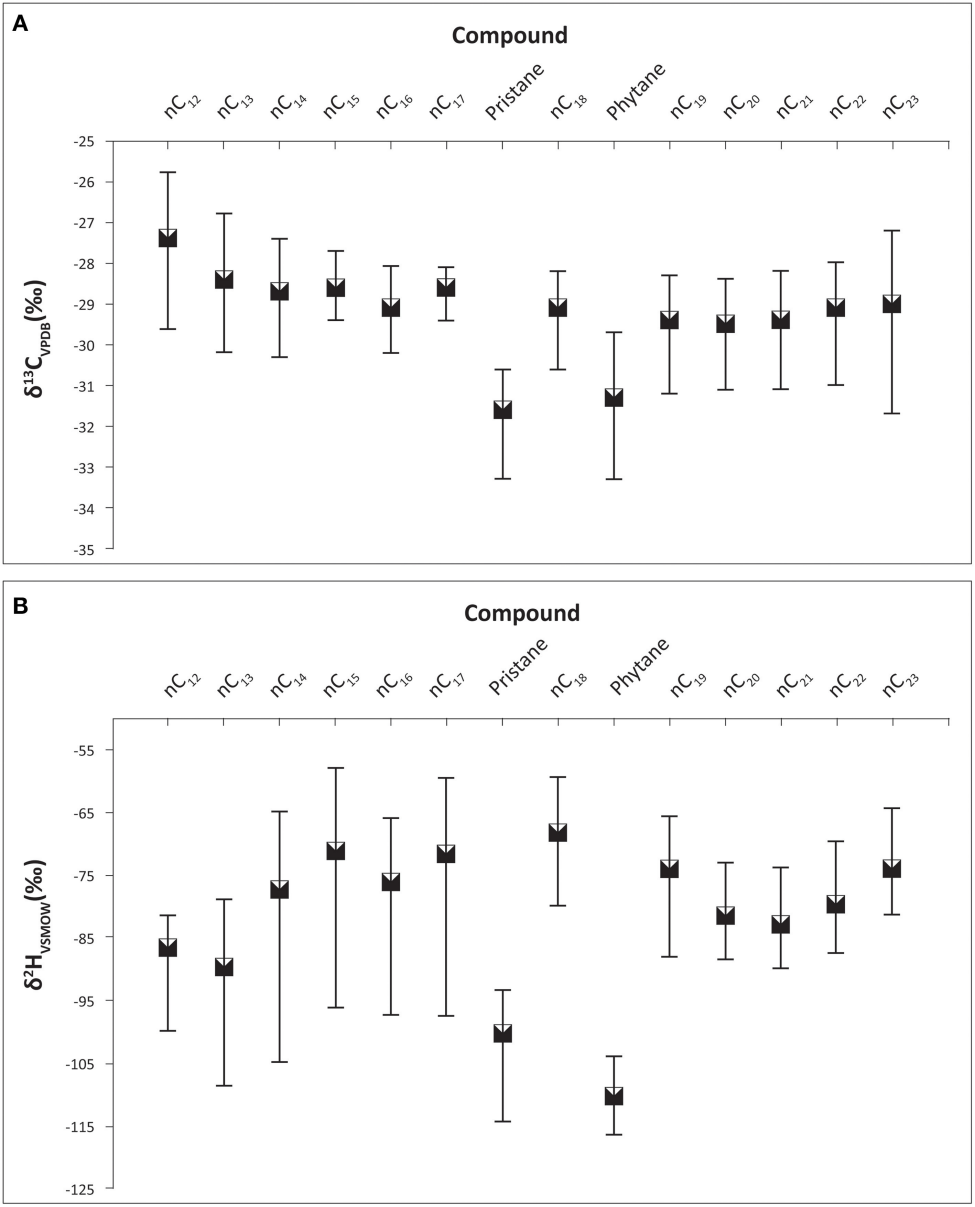
**Carbon and hydrogen isotope ratios of the alkane compounds found in Control, GKT, and South Island of New Zealand diesel samples**. Bars represent the range of isotope values for each compound. **(A)** The range of carbon isotope compositions of the alkanes within these diesel samples were between −33.3 to −25.8 ‰. **(B)** The range of hydrogen isotope compositions of the alkanes within these diesel samples were between −116.6 to −58.1 ‰.

The ranges of carbon isotope ratios of each individual alkane in Control, GKT and South Island of New Zealand samples are illustrated in Figure 2A. The mean value of pristane was lighter (−30.1%) when compared with phytane and the rest of the alkanes. Table 1 shows the statistical analysis of the δ^13^C values of individual alkanes in the same set of samples. The range of carbon isotope compositions of the alkanes within these diesel samples (−33.3 to −25.8%) was comparable to the values determined in previous work (Mazeas et al., 2002; Sun et al., 2005). These compounds show a broad range of carbon isotopic ratios, the greatest being observed for phytane (−33.3 to −28.3%), *n*C_23_ (−31.7 to −27.2%) and pristane (−33.3 to −29.2%). There was no significant difference in δ^13^C values of odd and even numbered alkanes.

**Table 1:** **Statistics of the δ^13^C and δ^2^H value of individual alkanes in Control, GKT, and South Island of New Zealand samples**.

	**N**	**Range**	**Minimum**	**Maximum**	**Mean**	***SD***	**|Variance**
*n*C_12_^13^C	56	3.9	−29.6	−25.8	−27.6	0.9	0.8
*n*C_13_^13^C	56	3.3	−30.2	−26.8	−28.4	0.9	0.8
*n*C_14_^13^C	56	2.8	−30.3	−27.4	−28.6	0.6	0.3
*n*C_15_^13^C	56	1.6	−29.4	−27.7	−28.6	0.4	0.1
*n*C_16_^13^C	56	2.3	−30.2	−27.9	−29.1	0.5	0.2
*n*C_17_^13^C	56	1.4	−29.5	−28.1	−28.6	0.4	0.1
Pris^13^C	56	4.1	−33.3	−29.2	−31.3	1.1	1.1
*n*C_18_^13^C	56	2.4	−30.6	−28.2	−29.1	0.6	0.4
Phy^13^C	56	4.9	−33.3	−28.3	−30.9	1.2	1.6
*n*C_19_^13^C	56	2.9	−31.2	−28.3	−29.3	0.7	0.5
*n*C_20_^13^C	56	2.7	−31.1	−28.4	−29.4	0.6	0.4
*n*C_21_^13^C	56	2.9	−31.1	−28.2	−29.4	0.8	0.6
*n*C_22_^13^C	56	3.0	−31.0	−28.0	−29.2	0.8	0.6
*n*C_23_^13^C	56	4.5	−31.7	−27.2	−29.1	1.2	1.3
*n*C_12_^2^H	56	54.6	−100.0	−45.5	−82.1	11.4	130.1
*n*C_13_^2^H	56	35.0	−108.9	−74.0	−88.4	7.9	62.7
*n*C_14_^2^H	56	39.9	−105.1	−65.1	−78.2	10.6	111.4
*n*C_15_^2^H	56	38.1	−96.2	−58.1	−72.8	11.3	126.7
*n*C_16_^2^H	56	31.3	−97.5	−66.2	−77.6	8.6	73.6
*n*C_17_^2^H	56	37.9	−97.6	−59.7	−73.3	11.0	122.0
Pris^2^H	56	29.2	−114.6	−85.4	−100.6	5.9	35.0
*n*C_18_^2^H	56	22.4	−82.1	−59.7	−69.8	6.0	36.3
Phy^2^H	56	39.1	−116.6	−77.5	−105.5	10.3	105.1
*n*C_19_^2^H	56	22.5	−88.3	−65.8	−74.3	4.8	23.2
*n*C_20_^2^H	56	19.8	−88.5	−68.7	−80.0	5.3	28.6
*n*C_21_^2^H	56	17.6	−90.0	−72.4	−82.3	4.8	22.8
*n*C_22_^2^H	56	19.1	−87.5	−68.5	−78.6	6.1	36.9
*n*C_23_^2^H	56	33.9	−86.4	−52.5	−73.7	6.8	46.4

### Hydrogen isotope analysis

Table 1 presents the δ^2^H values of individual alkanes in Control, GKT and South Island of New Zealand samples. The largest range of δ^2^H was found in *n*C_12_ (−100.0 to −45.5%), *n*C_15_ (−96.2 to −58.1%) and *n*C_17_ (−97.6 to −59.7%). The wide range of hydrogen isotopic values for these alkanes was in agreement with previous published result (Li et al., 2001). The ranges in δ^2^H values of each of the alkane compounds are illustrated in Figure 2B. The findings from the analysis of δ^2^H values revealed similar patterns as observed in the δ^13^C.

### Multivariate statistical analysis

The forensic evidence provided by stable isotope analysis is circumstantial in that when two samples are indistinguishable that supports, but does not prove, the hypothesis that they are related. The likelihood that they are actually related can only be estimated by measuring control samples and determining how likely two samples could have identical isotopic character by chance. In the present context it is important to establish whether samples obtained from the same source region have similar isotopic characteristics and are distinctive from samples obtained from other source regions. Therefore, it is imperative that control samples be subject to a population study in order for analysts to make an informed assessment of the likelihood. The control samples used here were commercially available diesel samples obtained from around the South Island of New Zealand (Muhammad et al., 2013) plus the Control samples supplied by NZ Police. The stable isotope ratios of individual alkanes from all the diesel samples were subjected to PCA and HCA. The information obtained from the statistical analyses was used to associate and differentiate diesel samples based on δ^13^C and δ^2^H values. The first principal component (PC1) described 50.4% of the variance in the data. The second principal component (PC2) described an additional 19.1% of the variance while the third principal component (PC3) explained further 9.6% of the data variability. Thus, 79.1% of the total variance was explained by the first three components.

Table 2 shows the component matrix which listed δ^13^C and δ^2^H values as variables and their contribution to the variance in the data set. Component matrix 1 shows the δ^13^C values of the alkane compounds contributed evenly with large positive coefficients. This implies that they have equal weighting in the interpretation of the scores for PC1. Likewise, δ^2^H values of the alkane compounds show positive coefficients for the same principal component. As for PC2, δ^13^C still shows positive coefficients for all the compounds. On the other hand, δ^2^H values of the compounds for this component show negative correlations to δ^13^C although both variables may have equal weighting and contribution to the interpretation of the scores. In component matrix 3, the top contributors to the scores were the δ^2^H values of *n*C_12_ and *n*C_18_ with highly negative coefficients.

**Table 2:** **Component matrix which shows variables and their contribution to the variance in the data set of Control and South Island of New Zealand samples**.

	**Component^a^**				
	**1**	**2**	**3**	**4**	**5**
*n*C_12_^13^C	0.728	0.445	0.153	−0.262	0.368
*n*C_13_^13^C	0.651	0.494	0.211	−0.184	0.324
*n*C_14_^13^C	0.713	0.454	−0.110	−0.253	0.304
*n*C_15_^13^C	0.144	0.774	0.216	0.323	0.268
*n*C_16_^13^C	0.264	0.678	0.302	0.478	0.176
*n*C_17_^13^C	0.457	0.479	0.520	0.301	−0.294
Pris^13^C	0.884	0.173	−0.079	0.268	0.182
*n*C_18_^13^C	0.827	0.429	−0.085	−0.103	−0.042
Phy^13^C	0.814	0.154	−0.338	0.242	0.089
*n*C_19_^13^C	0.869	0.358	−0.226	0.006	−0.101
*n*C_20_^13^C	0.754	0.412	−0.377	−0.066	−0.216
*n*C_21_^13^C	0.744	0.481	−0.298	0.013	−0.242
*n*C_22_^13^C	0.798	0.366	−0.227	0.117	−0.342
nC_23_^13^C	0.854	0.267	−0.124	0.123	−0.310
*n*C_12_^2^H	0.370	−0.386	−0.752	−0.077	0.191
*n*C_13_^2^H	0.794	−0.372	−0.093	−0.393	−0.016
*n*C_14_^2^H	0.876	−0.276	0.057	−0.324	−0.043
*n*C_15_^2^H	0.916	−0.287	0.085	−0.165	0.002
*n*C_16_^2^H	0.869	−0.229	0.175	−0.365	0.002
*n*C_17_^2^H	0.922	−0.224	0.131	−0.246	0.010
Pris^2^H	0.635	−0.378	0.316	0.026	0.008
*n*C_18_^2^H	0.686	−0.315	0.563	−0.061	−0.026
Phy^2^H	0.377	−0.427	−0.570	0.444	0.206
*n*C_19_^2^H	0.572	−0.607	0.333	0.228	0.130
*n*C_20_^2^H	0.673	−0.576	−0.098	0.266	0.029
*n*C_21_^2^H	0.579	−0.597	0.001	0.435	0.085
*n*C_22_^2^H	0.655	−0.549	0.019	0.325	−0.034
*n*C_23_^2^H	0.672	−0.305	0.488	0.107	−0.142

*Results are based on the δ^13^C and δ^2^H values of the alkane compounds*.

a *5 components extracted*.

**Figure 3:**
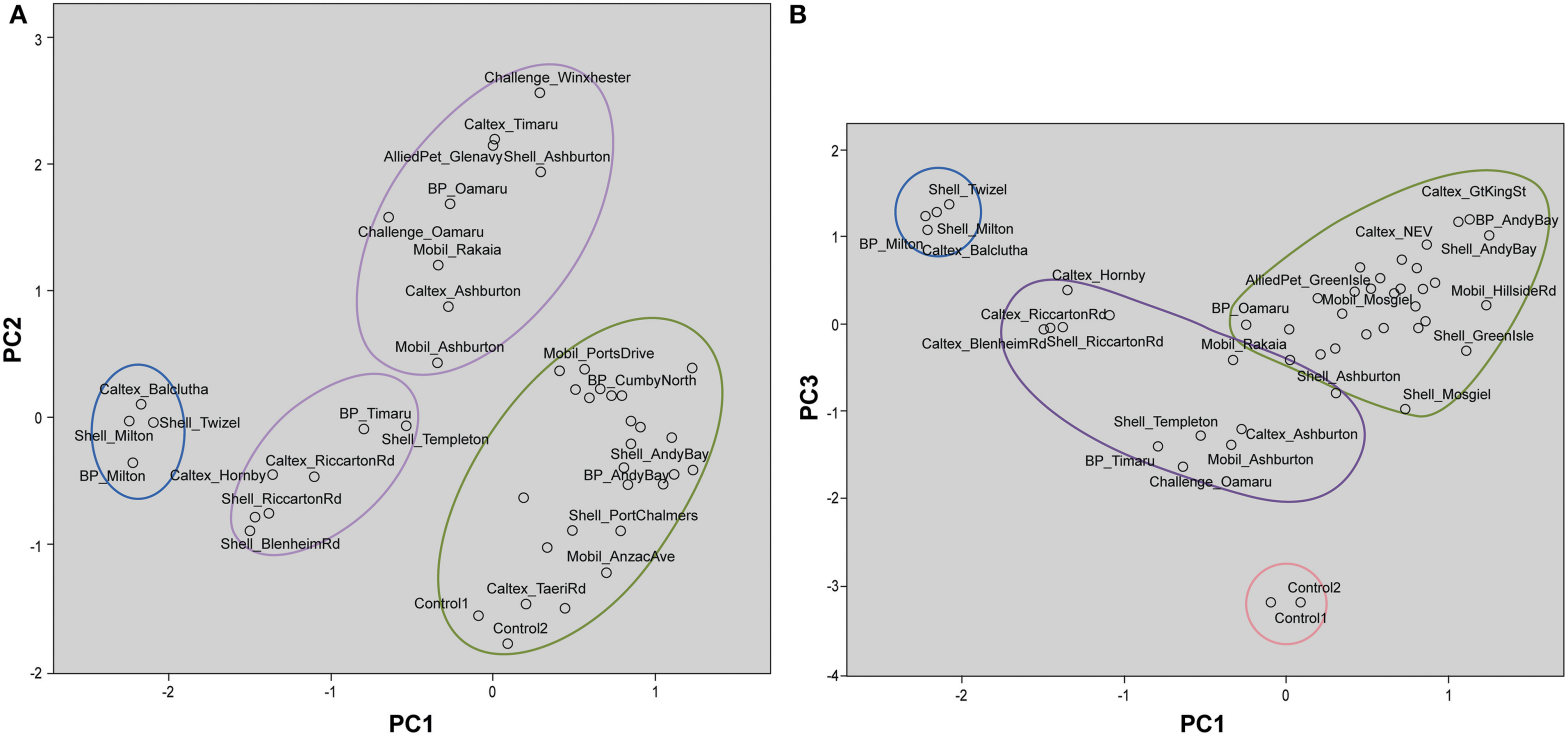
**Scores plot for principal component analysis**. It holds the scores for each sample on PC1 and PC2 **(A)**; PC1 and PC3 **(B)**. The names of some samples were removed for easier visualization. Distinct clustering can be seen for samples from Dunedin City (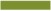), Canterbury region and North Otago (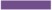), and South Otago and Twizel (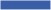). **(A)** Control samples were clustered among the samples from Dunedin City. **(B)** Control samples (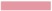) were clearly separated from the rest of the group by PC3.

These interpretations are visualized on the scores plot in Figure 3A (PC1 vs. PC2) and Figure 3B (PC1 vs. PC3). Figure 3A shows two distinct clusters with South Otago and Twizel samples in one group and Dunedin City in another. The Control samples are grouped in the same cluster as the Dunedin City samples. The samples obtained from the Canterbury and North Otago appears to be separated into 2 clusters by PC2. However, in Figure 3B several clusters are apparent comprising South Otago and Twizel samples in one group, Dunedin City in another and Canterbury region and North Otago samples in a group of its own in the middle of the plot. The Control samples were clearly separated from the other groups due to the substantial contribution by PC3.

**Figure 4:**
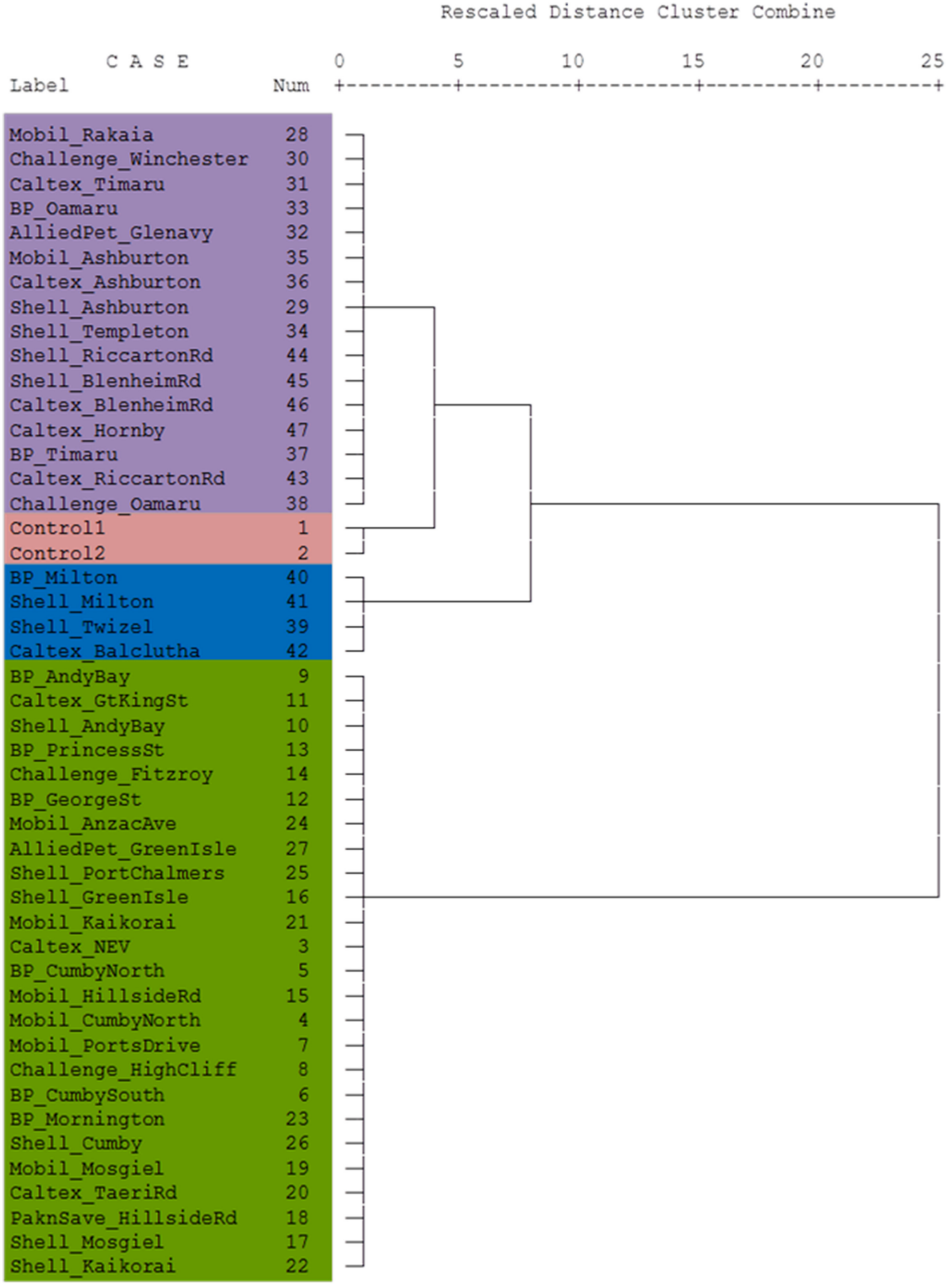
**Dendrogram using Ward's linkage method showing cluster relationship between Control and South Island of New Zealand**. Lengths of vertical lines represent statistical difference between multivariate components in each sample. The different colors reflect the different areas where the samples were obtained (see Figure 1).

The dendrogram presented in Figure 4 shows that the Control diesel samples were subtly correlated with the samples from Canterbury region and North Otago samples. South Otago and Twizel samples were grouped together and showed moderate correlation with the Control, Canterbury region and North Otago samples. The dendrogram also shows clear separation of Dunedin City samples from the rest of the group as illustrated in the large jump of the linkage which indicates the clusters are far apart (read to the right of the dendrogram to see the clusters).

The statistical analyses on the subtle differences in δ^13^C and δ^2^H of alkanes enabled clear discrimination of the Control samples from the other South Island samples. Following this observation, similar statistical treatments were carried out for Control and GKT samples, together with the South Island of New Zealand samples.

The PCA analysis resulted in a first principal component (PC1) that described 40.9% of the variance in the data. The second principal component (PC2) described an additional 17.9% of the variance while the third principal component (PC3) explained further 14.8% of the data variability. Thus, 73.6% of the total variance was explained by the first three components.

Table 3 shows the component matrix with δ^13^C and δ^2^H values as variables and their contribution to the variance in the data set comprised of the Control, GKT and South Island of New Zealand samples. Component matrix 1 shows the δ^13^C and δ^2^H values of the alkane compounds contributed evenly with highly positive coefficients for most of the compounds. This implies that they have equal weighting in the interpretation of the scores for PC1. For PC2, δ^2^H values of the compounds were mostly negatively correlated to the δ^13^C variables with more compounds showing higher weightage and contributed more to the interpretation of the scores. In component matrix 3, an opposing trend for δ^13^C and δ^2^H values was seen with δ^13^C variables showing negative correlations to that of δ^2^H variables.

**Table 3:** **Component matrix which shows variables and their contribution to the variance in the data set of Control, GKT, and South Island of New Zealand samples**.

	**Component^a^**				
	**1**	**2**	**3**	**4**	**5**
*n*C_12_^13^C	0.709	−0.199	−0.486	0.012	0.008
*n*C_13_^13^C	0.686	0.009	−0.437	−0.064	0.135
*n*C_14_^13^C	0.751	0.202	−0.303	−0.176	−0.148
*n*C_15_^13^C	0.254	0.364	−0.626	−0.241	0.435
*n*C_16_^13^C	0.335	0.644	−0.308	−0.071	0.527
*n*C_17_^13^C	0.481	0.078	−0.456	0.226	0.544
Pris^13^C	0.738	0.541	0.202	−0.203	0.180
*n*C_18_^13^C	0.863	0.172	−0.312	0.045	−0.086
Phy^13^C	0.663	0.603	0.249	−0.243	0.015
*n*C_19_^13^C	0.878	0.364	−0.102	−0.034	−0.151
*n*C_20_^13^C	0.773	0.452	−0.144	−0.002	−0.273
*n*C_21_^13^C	0.757	0.050	−0.404	0.151	−0.302
*n*C_22_^13^C	0.774	0.069	−0.284	0.370	−0.219
nC_23_^13^C	0.812	0.027	−0.185	0.419	−0.133
*n*C_12_^2^H	0.200	0.724	0.558	0.054	−0.218
*n*C_13_^2^H	0.715	0.049	0.431	−0.264	−0.203
*n*C_14_^2^H	0.810	−0.448	0.201	−0.148	−0.144
*n*C_15_^2^H	0.788	−0.561	0.138	−0.065	−0.057
*n*C_16_^2^H	0.777	−0.555	0.080	−0.149	−0.101
*n*C_17_^2^H	0.799	−0.571	0.076	−0.025	−0.076
Pris^2^H	0.487	−0.439	0.105	−0.529	0.208
*n*C_18_^2^H	0.491	−0.792	−0.011	0.049	0.262
Phy^2^H	0.215	0.674	0.552	−0.370	0.061
*n*C_19_^2^H	0.447	−0.565	0.455	−0.027	0.384
*n*C_20_^2^H	0.539	0.000	0.667	−0.230	0.159
*n*C_21_^2^H	0.389	−0.020	0.643	0.463	0.222
*n*C_22_^2^H	0.457	0.130	0.644	0.479	0.158
*n*C_23_^2^H	0.382	0.210	0.261	0.634	0.199

*Results are based on the δ^13^C and δ^2^H values of the alkane compounds*.

a *5 components extracted*.

**Figure 5:**
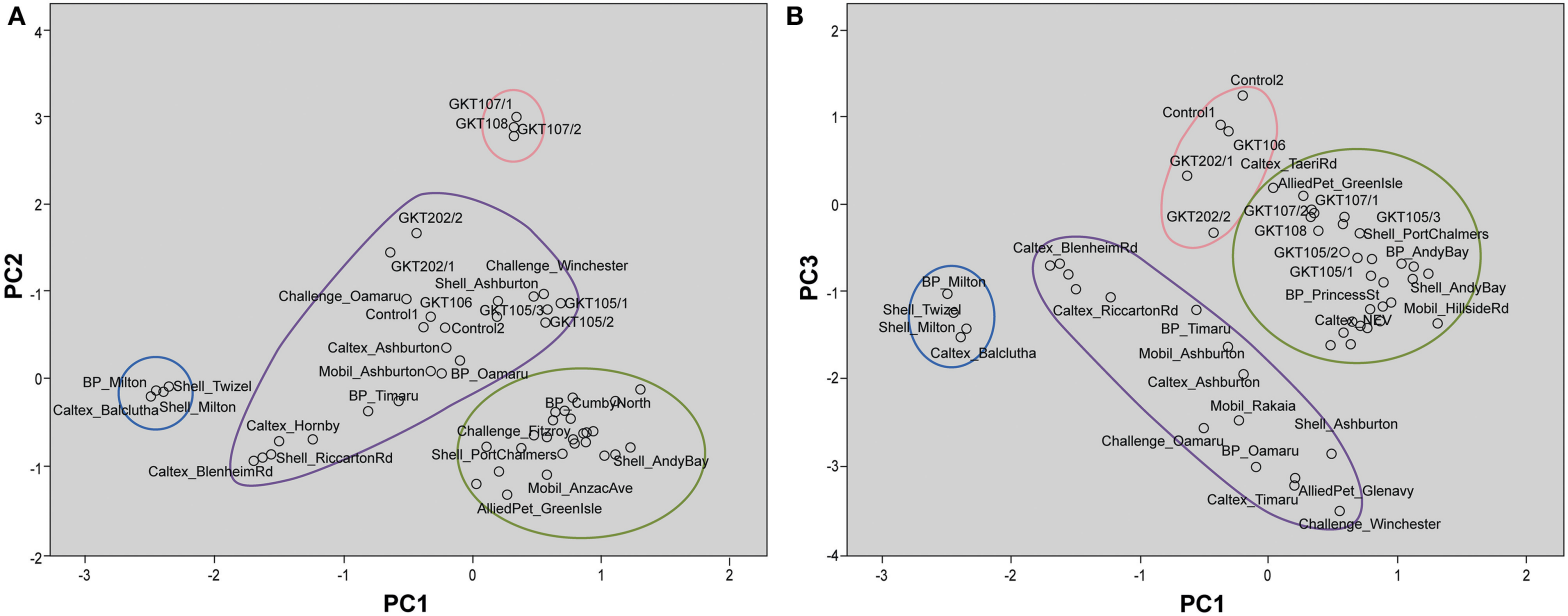
**Scores plot for principal component analysis**. It holds the scores for each sample on PC1 and PC2 **(A)**; PC1 and PC3 **(B)**. The names of some samples were removed for easier visualization. **(A)** Distinct clustering can be seen for samples from Dunedin City (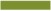), Canterbury region and North Otago (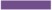), and South Otago and Twizel (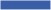). GKT 107/1, GKT107/2, and GKT 108 (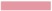) were separated from the rest of the groups by PC2 whilst Control and other GKT samples were grouped in the cluster of Canterbury region and North Otago. **(B)** Distinct clustering can be seen for samples from Dunedin City (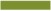), Canterbury region and North Otago (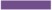), and South Otago and Twizel (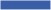). PC3 showed substantial contribution in separating Control, GKT 106, GKT 202/1, and GKT 202/2 samples (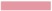) from the rest of the data set.

These interpretations from PCA were visualized on the scores plots in Figures 5A,B. Figure 5A shows distinct clusters comprised of South Otago and Twizel samples in one group and Dunedin City samples in another. The Canterbury region and North Otago samples showed moderate clustering in the middle of the scores plot. GKT 107/1, GKT 107/2, and GKT 108 were separated from the rest of the diesel samples by PC2 whilst Control and other GKT samples fell in the cluster of Canterbury region and North Otago samples. When PC1 was plotted against PC3 (Figure 5B), similar observations were seen for South Otago and Twizel and Dunedin City clustering in Figure 5A. The primary difference is that Control samples are now grouped together with GKT 106, GKT 202/1, and GKT 202/2. Clearly, this pattern was mainly due to the contribution from PC3. However, some of the GKT samples were seen to have similarities with that of Dunedin City samples as scores plot showed moderate clustering between them.

**Figure 6:**
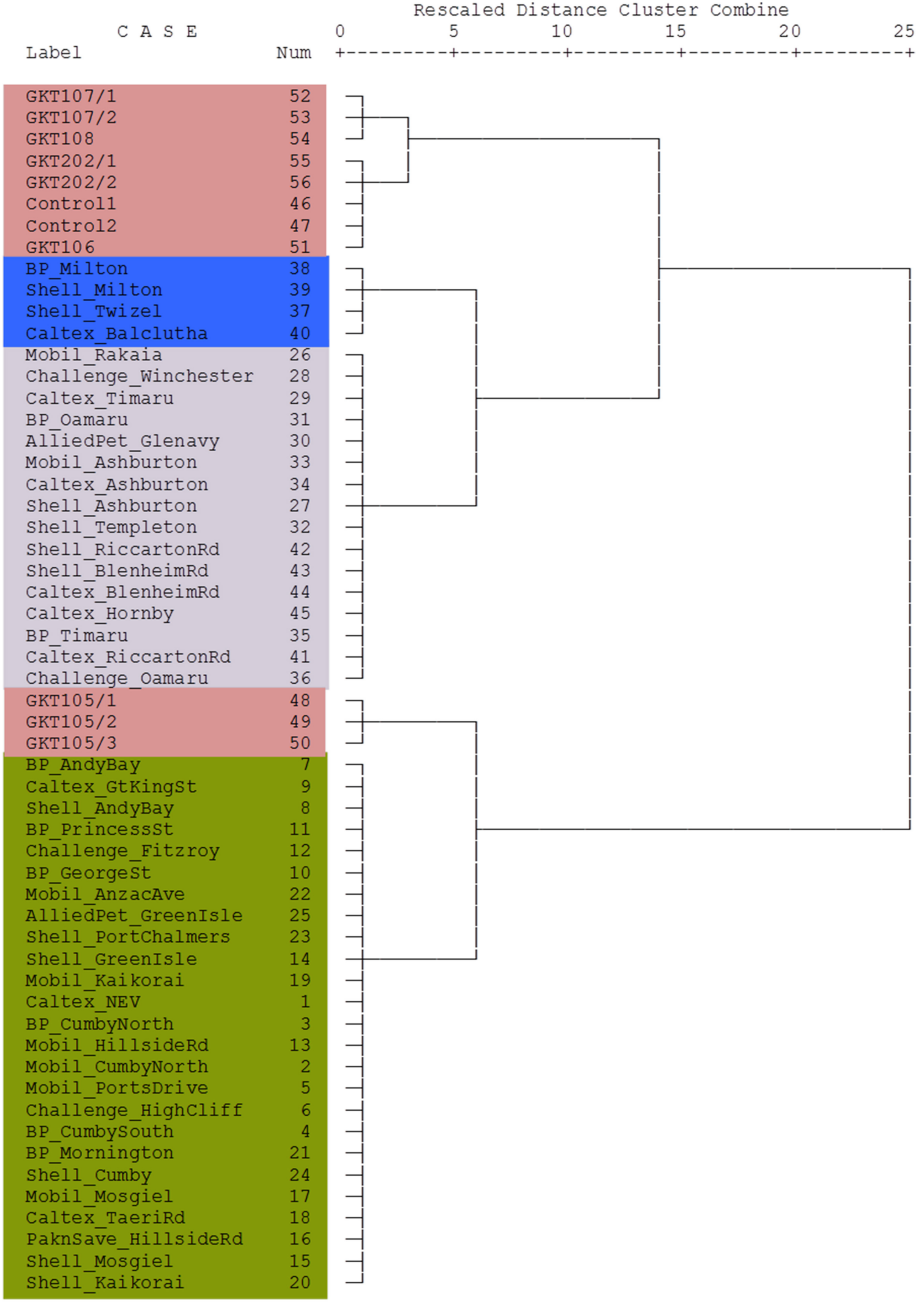
**Dendrogram using Ward's linkage method showing cluster relationship between Control, GKT, and South Island of New Zealand samples**. Lengths of vertical lines represent statistical difference between multivariate components in each sample. The different colors reflect the different areas where the samples were obtained (see Figure 1).

The stable isotope data of the diesel samples involved in the theft case were also analyzed using cluster analysis to see their association with the diesel samples from the South Island of New Zealand. This relationship was presented in a dendrogram in Figure 6. Here the Blenheim diesel samples are grouped into small clusters with samples under the same denominations such as GKT 107 samples and GKT 108, classed together. Likewise, the GKT 106 sample found to be highly correlated with GKT 202 samples and Control diesels. Additionally, except for GKT 105 samples, the rest of the Blenheim diesels were closely related and showed only a small amount of variation between them. GKT 105 diesels were more closely correlated with the samples from Dunedin City service stations than the samples from Blenheim, as seen by the large jump of the linkage between them. Similar discrimination is observed for the rest of the commercial diesel samples with each of the group classed together due to the high correlation between them.

## Conclusions

The forensic discrimination of diesel fuels involved in a theft case utilizing stable isotope fingerprint of hydrocarbon indices was achieved. There were variations in the stable isotopic compositions of alkanes within the diesel samples that can be used to differentiate them. Additionally, a large population of commercial diesel fuels obtained from various areas in the South Island of New Zealand was included in the data set to rule out statistical coincidence as well to put the data into context thus providing an informed assessment of the analysis.

Discrimination of the diesel samples into groups was evident by comparing the subtle differences in the stable isotope values of the alkane compounds using multivariate statistical analyses such as PCA and HCA. The main conclusions from these analyses are:

- PCA showed that the Control diesel samples were strongly correlated with GKT 106 and GKT 202 samples and moderately correlated with the other GKT samples.
- Diesel samples that were grouped together based on PCA also showed high correlation using HCA.
- However, GKT 105 samples were shown to be grouped separately from the other Blenheim samples using HCA.
- PCA and HCA both highlighted the association of Control samples with that of GKT 106, GKT 107, GKT 108, and GKT 202 samples.
- In our opinion, the most likely explanation is that these diesels share a common history.

In summary, this paper provided some context on the Blenheim diesel theft case. The Control samples were found to be chemically related to samples found in possession of the suspect, i.e., they were indistinguishable by measurements of their stable isotope fingerprint. That they were distinguishable from the 45 additional samples collected from around the South Island and that the South Island samples were able to be grouped according to source provided support for the prosecution hypothesis that the samples in possession of the suspect had originated from the theft. When this evidence was presented in court the accused changed their plea to guilty and was convicted of the theft.

### Conflict of interest statement

The authors declare that the research was conducted in the absence of any commercial or financial relationships that could be construed as a potential conflict of interest.
